# Age at adiposity rebound in childhood is associated with PCOS diagnosis and obesity in adulthood—longitudinal analysis of BMI data from birth to age 46 in cases of PCOS

**DOI:** 10.1038/s41366-019-0318-z

**Published:** 2019-02-04

**Authors:** E. Koivuaho, J. Laru, M Ojaniemi, K. Puukka, J. Kettunen, J. S. Tapanainen, S. Franks, M.-R. Järvelin, L. Morin-Papunen, S. Sebert, T. T. Piltonen

**Affiliations:** 1 Department of Obstetrics and Gynaecology, PEDEGO Research Unit, Medical Research Center, University of Oulu and Oulu University Hospital, Oulu, Finland; 2 Department of Children and Adolescents, PEDEGO Research Unit, Medical Research Center, University of Oulu and Oulu University Hospital, Oulu, Finland; 3 NordLab Oulu, Department of Clinical Chemistry, Medical Research Center, University of Oulu and Oulu University Hospital, Oulu, Finland; 4 Computational Medicine, Faculty of Medicine, University of Oulu, Oulu, Finland; 5 Biocenter Oulu, University of Oulu, Oulu, Finland; 6 Department of Obstetrics and Gynaecology, University of Helsinki and Helsinki University Hospital, Helsinki, Finland; 7 Institute of Reproductive and Developmental Biology, Department of Surgery & Cancer, Imperial College London, London, UK; 8 Center for Life Course Health Research, University of Oulu, Oulu, Finland; 9 Department of Epidemiology and Biostatistics, MRC-PHE Centre for Environment and Health, School of Public Health, Imperial College London, London, UK; 10 Unit of Primary Care, Oulu University Hospital, Oulu, Finland; 11 Department of Genomic of Complex Diseases, School of Public Health, Imperial College London, London, UK

**Keywords:** Obesity, Epidemiology, Risk factors, Obesity, Paediatrics

## Abstract

**Background:**

Adiposity rebound (AR), the second BMI rise in childhood at around the age of 6 years, is associated with obesity and metabolic alteration in later life. Given that polycystic ovary syndrome (PCOS) has a strong metabolic component, early life growth patterns could reveal a risk of PCOS. Thus, we aimed to investigate the associations between age at AR and PCOS diagnosis and BMI later in life.

**Materials and methods:**

This study is part of a prospective, population-based longitudinal study, where women with PCOS diagnosis by age 46 (*n* = 280) were compared with asymptomatic women (CTRLs, *n* = 1573). Weight and height data from birth to age 13 years, at age at menarche, and at ages 31 and 46 years were analyzed

**Results:**

Women with PCOS had lower birth weight (3357 ± 477 vs. 3 445 ± 505 g, *p* < 0.001), earlier age at AR (5.2 ± 1.0 vs. 5.6 ± 0.90 years, *p* < 0.001) and higher BMI from AR onwards compared with controls. Early timing of AR was associated with PCOS diagnosis independently of BMI (OR 1.62, 95% Cl 1.37–1.92). Women with PCOS and early AR had higher BMI at 31 and 46 years when compared to controls with early AR. The age at AR did not associate with T levels at ages 31 or 46 years.

**Conclusions:**

Early AR was associated with PCOS diagnosis and high BMI in adulthood. Adolescent girls with early AR and persisting obesity should be screened for PCOS symptoms, such as persistent irregular cycles and hirsutism.

## Introduction

Childhood obesity is an epidemic problem worldwide, often leading to adult obesity and early occurrence of several obesity-related conditions such as type 2 diabetes and metabolic syndrome [1]. Recently, prediction modeling revealed that almost 60% of children in the United States will be obese at the age of 35 years, with half already being obese during childhood [2].

There is consistent evidence that birth weight is associated with cardiovascular disease risk factors later in life, especially in children born small or large for gestational age (SGA/LGA) [3, 4]. Childhood body mass index (BMI) growth trajectory data have also shown its value as a tool to estimate BMI and metabolic risks in adulthood [5]. Indeed, early timing of adiposity rebound (AR), the second rise in BMI following a nadir occurring in early childhood, has been associated with increased risks of obesity and metabolic derangements both in adolescence and in adulthood [5, 6]. The mean age at AR is population-dependent and occurs at 5–6 years, according to population-based BMI trajectories [5]. According to the literature, early timing of AR is defined as that occurring before the age of 5 years [5].

Polycystic ovary syndrome (PCOS) is a multifactorial disorder that affects up to 18% of women of reproductive age. It is associated with high-level morbidity and is an economic burden to society [7]. Affected women suffer from hyperandrogenism, anovulation, infertility, and adverse cardio-metabolic and/or psychological outcomes, those with hyperandrogenism presenting with most adverse BMI outcomes [8–10]. Although the syndrome is typically identified during the reproductive period, it seems to originate as early as in pre-pubertal years or even during the prenatal period when prenatal exposure to androgen excess could predispose to PCOS [11, 12]. More than a half of women with PCOS are obese, and later, obesity amplifies the symptoms of PCOS, especially if the weight gain has occurred in early adulthood [9, 13, 14]. Given that there is a pandemic rise in pre-pubertal obesity, more pronounced weight gain in childhood could be a triggering factor of PCOS. In support of this hypothesis, a recent study by Zegher et al. showed early deviation in growth patterns in obese adolescents with PCOS [15]. However, the study did not elucidate the role of AR in the diagnosis of PCOS. In fact, in the current literature, childhood obesity and the risk of PCOS has received little interest so far.

As women with PCOS are generally accepted to be at a high risk of obesity and adverse cardio-metabolic profiles, as well as having several other comorbidities, it would be of utmost importance to identify these individuals at risk early on in order to allow possible intervention and support. The present study was thus designed to identify early risk factors for PCOS in a nested case–cohort study. We especially tested whether birth weight and the timing of the AR could influence the risk of PCOS diagnosis by age 46. Second, we aimed to analyze whether early timing of AR could associate with higher BMI or testosterone levels later in life in women with PCOS.

## Materials and methods

### The Northern Finland Birth Cohort 1966

The study population is part of the prospective, longitudinal, population-based, Northern Finland Birth Cohort (NFBC) 1966, recruited at gestational week 24 from the two northernmost provinces of Finland. The study concerns a total of 12,058 live births (5889 females), covering 96% of all births in this area. Since birth there have been four follow-up time points with postal questionnaires and/or clinical examinations at ages 1, 14, 31, and 46 years. The study was approved by the Ethics Committee of the Northern Ostrobothnia Hospital District. All participants provided an informed consent.

At the age of 31 years old a postal questionnaire was sent to 5608 women and 4523 answered (81% response rate). The participants were asked questions on lifestyle, occupation and working history, living environment, and health. In addition, 3127 (76%) women participated in clinical examinations including fasting blood sampling and anthropometric measurements.

At age 46, all women participants with a known address in Finland were invited to participate to a follow-up. A total of 5123 women received a postal questionnaire and 3706 (72%) answered. Clinical examinations with blood samples and anthropometric measurements were performed in 3280 (64%) women. The study flow chart is presented in Supplementary Figure 1.

### Identification of women with PCOS

The 31-year follow-up questionnaire included two questions screening for PCOS symptoms: (i) is your menstrual cycle longer than 35 days more than twice a year (oligomenorrhea, OA); and (ii) do you have bothersome, excessive body hair (hirsutism, H)? Both symptoms were reported in 4.2% (*n* = 125) and they were considered to fulfill the criteria for PCOS. The validity of identifying women with PCOS by way of these two questions has been established in previous publications [9, 16]. In the 46-year follow-up questionnaire the women self-reported whether they had been diagnosed with polycystic ovaries (PCOs) or PCOS (“Have you ever been diagnosed as having polycystic ovaries and/or polycystic ovary syndrome (PCOS)?”), and 181 women answered “yes” to this question. The total PCOS group was assembled from women reporting both OA + H at age 31 and/or self-reporting PCOs/PCOS diagnosis at age 46 (referred to as the total PCOS group, tPCOS; *n*_total_ = 280). We excluded women using hormonal contraceptives and pregnant women (*n* = 1488), and women not permitting use of their data (*n*_31_ = 41 and *n*_46_ = 14). The control group (CTRLs) included women without any PCOS symptoms at age 31 and without PCOS diagnosis by age 46 (*n*_total_ = 1573) (Supplementary Fig. 1).

### Birth weight and AR

Data on birth weight was collected prospectively by local midwives in the antenatal clinics and the data were available for all the cohort members. Similarly, gestational age was defined from the last menstrual period and the data were collected by the midwives. Individuals with birth weight under the 10th percentile for gestational age were considered as SGA and individuals with birth weight over the 90th percentile for gestational age were considered as LGA.

Infancy and childhood growth measurements (weight and height from early infancy to adolescence) were gathered from the welfare clinic records. The measurements were collected by nurses at welfare clinics as part of the national child-health screening program that is free and available for all children born in Finland. Modeling of growth in infancy (2 weeks to 18 months) and childhood (18 months to 13 years) was carried out separately. There were approximately 7 measurements in infancy and 16 measurements in childhood per subject. Early BMI data (weight and height) were available for 1010 (64.2%) controls, and 189 (67.5%) women with PCOS. Longitudinal modeling, derivation of BMI in infancy and childhood, and timing of the adiposity peak (AP) and AR were calculated from fitted BMI curves as described previously [18]. Having clinical implications in mind, we set an age cutoff value for early AR by dividing the study population into quartiles according to age at AR. The early AR quartile limit value was ≤5.1 years in the present female population, being in line with previously published data. As late timing of AR has not been reported to be associated with adverse metabolic outcomes, other quartiles (children with AR > 5.1 years) were pooled together to represent women with normal/late AR.

### Covariates

#### BMI and waist circumference at menarche and/or at ages 31 and 46

In the 31-year postal questionnaire the women reported their age at menarche. BMIs at menarche were estimated from the fitted BMI curves.

In the clinical examinations during cohort data collection, weight was measured using a digital scale and height was measured twice using a standard calibrated stadiometer. BMI (kg/m^2^) was calculated using measured weight and height (average of two measurements). If clinical measurements were missing, self-reported values of weight and height were utilized. There was no statistically significant difference between self-reported and clinically measured BMIs. Waist circumference (WC; cm) at ages 31 and 46 was measured midway between the lowest rib margin and the iliac crest. Changes in BMI were calculated from age 14 to age 31, from age 31 to age 46, and from age 14 to age 46.

#### Maternal variables

For covariate analysis, maternal pre-pregnancy BMI, maternal smoking, and gestational age at birth were utilized. The data on gestational age were collected as described above. Information on maternal age, education, pre-pregnancy BMI, and smoking status during pregnancy was self-reported and data were collected by local midwives in the antenatal clinics using a questionnaire that was designed for NFBC data collection.

#### Laboratory methods

Blood samples were drawn at ages 31 and 46. Serum testosterone (T) and sex hormone-binding globulin (SHBG) were analyzed as described earlier [17]. Briefly, T were analyzed with liquid chromatography/mass spectrometry equipment with an electrospray ionization source operating in positive-ion mode (Agilent Technologies, Inc., Wilmington, DE, USA). At age 31 SHBG was assayed by fluoroimmunoassay (Wallac, Inc. Ltd., Turku, Finland) and at age 46 by chemiluminometric immunoassay (Immulite 2000, Siemens Healthcare, Llanberis, UK). Because of a level difference in the assays used at different time points, values at age 31 were amended as follows: (0.7615 × SHBG at age 31) + 0.7088 nmol/l. The free androgen index (FAI) calculation: 100 × T (nmol/l)/SHBG (nmol/l). In addition, at age 46 calculated free testosterone (cFT) was assessed by using albumin, SHBG, and testosterone concentrations, according to the method described by Vermeulen et al. [18].

### Statistical methods

IBM SPSS Statistics version 22 for Windows (SPSS, Inc., 1989, 2013, IBM Corp.) was used to assess differences between the study groups and for performing the regression analyses. RStudio version 3.3.2 was used for longitudinal modeling and derivation of BMI trajectories from birth to 17 months and from 18 months to 13 years of age. Modeling was carried out separately as regards infancy and childhood. Briefly, the longitudinal BMI linear mixed-effect model was fitted using logarithmically transformed BMI as the outcome and the predicted timing of AP and AR was calculated using estimated fixed and random coefficients [19].

Differences in continuous variables (anthropometric parameters, AR timing, and hormonal outcomes) were analyzed by using the independent samples *t*-test, the Mann–Whitney *U* test, one-way analysis of variance or the Kruskal–Wallis test, as appropriate, and for differences in categorical parameters, the Chi-square test was utilized. For these tests, the results are reported as means with standard deviation or medians with 25% and 75% quartiles, and prevalence (%). The association between AR timing and PCOS was analyzed by using binary logistic regression modeling. Covariate analysis was also performed by including several variables in different models. The results are reported as odds ratios (ORs) with 95% confidence intervals. A value of *p* < 0.05 was considered statistically significant.

## Results

### Birth weight and gestational age

**Fig. 1:**
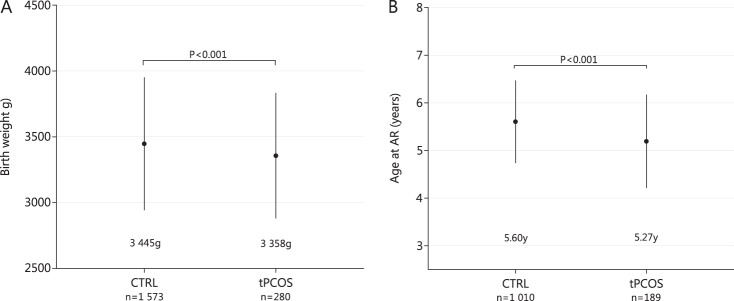
Birth weight (**a**) and adiposity rebound (AR) age (**b**) in control women (CTRL) and women with polycystic ovary syndrome (PCOS) by age 46 (tPCOS). Women with PCOS had lower birth weight and age at AR was lower in these women compared with controls. Significance assessed by using one-way analysis of variance and the Games-Howell post hoc test

The women with PCOS had lower birth weight compared with controls (Fig. 1a, Table 1). As for gestational age and the number of children born LGA or SGA and children <2500 g, no differences were found between the study groups (Table 1). However, there were more women with PCOS born preterm compared with controls (Table 1).

**Table 1:** Population characteristics

		Control women (*n* = 1573)^a^	tPCOS (*n* = 280)^a^	*p* ^b^
Gestational age (weeks)		39 ± 7	38 ± 8	0.221
Prematurity (born before 37th GW)		7.8%	11.4%	0.040
Birth weight (g)		3445 ± 505	3357 ± 477	0.006
	≤ 2500 g	3.8%	3.2%	0.660
Size for gestational age				0.410
	SGA	8.6%	7.8%	
	AGA	79.9%	83.2%	
	LGA	11.5%	9.0%	
Age at AP (months)		9.1 ± 0.4	9.1 ± 0.4	0.539
BMI at AP (kg/m^2^)		17.8 ± 0.8	17.7 ± 0.9	0.232
Age at AR (years)		5.6 ± 0.9	5.2 ± 1.0	<0.001
BMI at AR (kg/m^2^)		15.3 ± 1.1	15.6 ± 1.5	0.004
Age at menarche (years)		12.9 ± 1.2	12.7 ± 1.4	0.336
BMI at menarche (kg/m^2^)		17.9 (16.7; 19.3)	18.9 (17.6; 20.5)	<0.001
BMI at 31 y (kg/m^2^)		22.6 (20.7; 24.9)	24.2 (21.7; 28.3)	<0.001
BMI at 46 y (kg/m^2^)		25.3 (22.7; 29.1)	27.3 (24.2; 31.9)	<0.001
WC at 31 y (cm)		76.0 (70.5; 83.4)	81.5 (73.0; 92.1)	<0.001
WC at 46 y (cm)		84.0 (77.0; 94.0)	88.5 (81.0; 99.1)	<0.001
Smoking 31 y				0.260
	Non-smoker	51.2%	50.0%	
	Former/occasional smoker	26.4%	23.3%	
	Active smoker	22.4%	26.7%	
Smoking 46 y				0.280
	Non-smoker	56.3%	54.0%	
	Former/occasional smoker	25.6%	23.6%	
	Active smoker	10.1%	22.4%	
Education 31 y				0.519
	Basic	8.5%	12.9%	
	Secondary	73.7%	73.4%	
	Tertiary	17.8%	13.7%	
Education 46 y				0.519
	Basic	8.5%	12.9%	
	Secondary	63.4%	65.1%	
	Tertiary	31.1%	27.0%	
Testosterone 31 y (nmol/l)		0.96 (0.75; 1.25)	1.29 (0.93; 1.72)	<0.001
Testosterone 46 y (nmol/l)		0.82 (0.63; 1.05)	0.88 (0.68; 1.11)	0.017
SHBG 31 y (nmol/l)		60.0 (45.0; 81.6)	50.8 (33.4; 75.1)	<0.001
SHBG 46 y (nmol/l)		54.3 (38.0; 74.9)	49.3 (34.2; 66.7)	0.011
FAI 31 y		1.59 (1.12; 2.22)	2.75 (1.50; 4.10)	<0.001
FAI 46 y		0.02 (0.01; 0.02)	0.02 (0.01; 0.03)	<0.001
cFT 46 y (nmol/l)		0.01 (0.008; 0.014)	0.012 (0.010; 0.015)	<0.001

Data reported as mean ± SD or median with 25% and 75% quartiles, and as percentiles. Significance tests for continuous variables were performed by using the independent samples *t*-test or the Mann–Whitney *U* test, as appropriate, and for categorical variables we used Pearson’s test. *p*-Value < 0.05 considered significant

*AP* adiposity peak, *AR* adiposity rebound, *BMI* body mass index, *WC* waist circumference, *SHBG* sex hormone-binding globulin, *FAI* free androgen index, *cFT* calculated free testosterone, *PCOS* polycystic ovary syndrome, *SGA* small for gestational age, *AGA*appropriate for gestational age, *LGA* large for gestational age

^a^Differences in numbers vary in different analyses as a result of some missing data

^b^*p*-Values are for women with PCOS compared with control women

### Early BMI growth trajectory data and timing of AR in women with PCOS

**Fig. 2:**
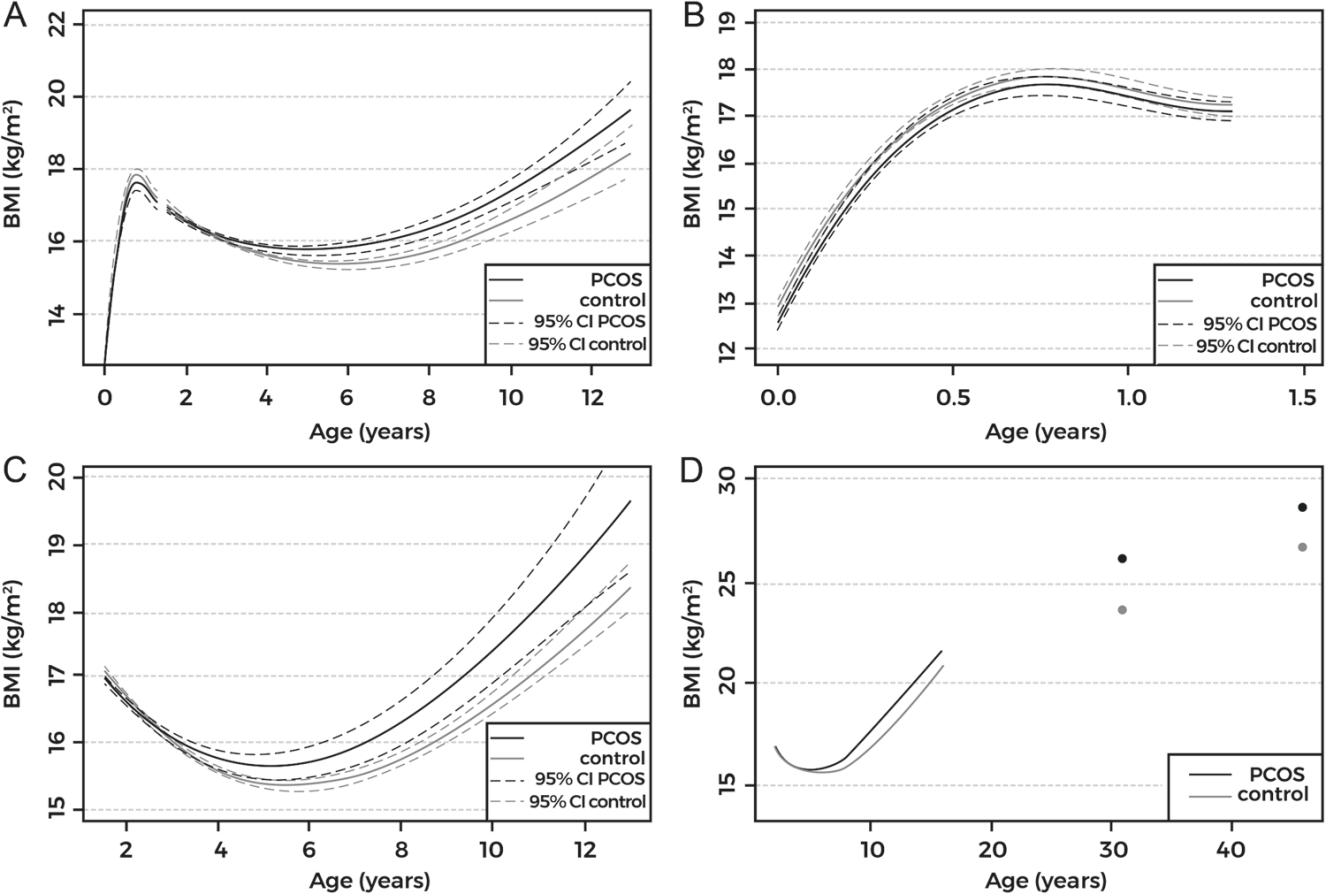
Body mass index (BMI) trajectories in women with polycystic ovary syndrome (tPCOS, black line) and in controls (gray line). 95% confidence intervals (95% CI) shown as dashed line in **a**–**c**. BMI trajectories from birth to 13 years show early adiposity rebound (AR) in women with PCOS (**a**). No difference was observed in the growth patterns during infancy (from birth until 18 months, including adiposity peak) (**b**). Detailed childhood growth pattern analysis from 18 months until 13 years showed early AR in women with PCOS (**c**). BMI trajectory from 18 months to 13 years and single BMI data points at ages 31 and 46 (**d**). Women with PCOS present with early deviation in growth pattern (BMI) from AR onwards, extending to adulthood

We observed no differences between PCOS and control groups at AP, whereas the longitudinal childhood BMI trajectory data revealed earlier AR timing and higher BMIs in women with PCOS compared with controls from AR timing onwards (Figs. 1b and 2, Table 1). Furthermore, women with PCOS were overrepresented in the earliest AR quartile (PCOS vs. CTRLs: 45.0% vs. 25.0%, *p* < 0.001).

### Associations of birth weight and AR with PCOS by age 46

**Fig. 3:**
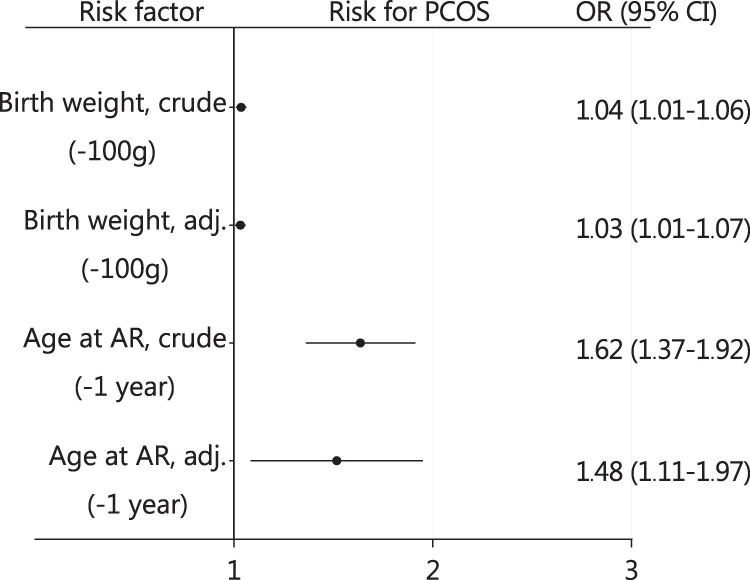
Association between birth weight and timing of adiposity rebound (AR) with polycystic ovary syndrome (PCOS) diagnosis by age 46. The result are expressed, for birth weight: by 100 g decrease in birth weight and for AR per 1 year decrease in the age at AR. Hosmer and Lemeshow GOF test *p*_AR_ = 0.389 and *p*_BW_ = 0.144. The analyses were carried out using logistic regression and the results are reported as odds ratios (ORs) with 95% confidence intervals (95% CIs). Birth weight was adjusted for maternal pre-pregnancy body mass index (BMI), maternal smoking, and gestational age. Timing of AR was adjusted for maternal smoking, pre-pregnancy BMI, gestational age, BMI at ages 31 and 46, waist circumference, and testosterone at age 31. Adjustment models shown in Supplementary Figure 3

In both adjusted and unadjusted models, logistic regression analysis revealed lower birth weight and early timing of AR to be risk factors of PCOS, although the risk related to AR seemed to be higher (Fig. 3). The women reporting OA + H and presenting more severe PCOS phenotype had even earlier AR compared to the srPCOS (4.96 years ± 1.11 vs. 5.27 ± 0.90, *p* = 0.010). In women with PCOS the association with early AR remained once adjusted for BMI, WC, and serum T (Fig. 3).

### BMI at menarche and at ages of 31 and 46 in PCOS and control groups

**Fig. 4:**
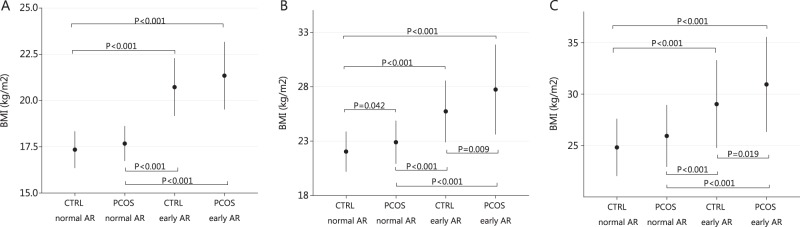
Body mass index (BMI) at menarche (**a**) and at the ages of 31 (**b**) and 46 (**c**) in women with polycystic ovary syndrome (PCOS) and in controls with early adiposity rebound (AR) and normal/late AR. Women with early AR had higher BMI at menarche and at ages 31 and 46, particularly those with PCOS. Significance assessed by using the Kruskal–Wallis test with multiple comparisons

Age at menarche did not differ between the study groups (Table 1). However, the women with PCOS had higher BMI at menarche, compared with controls (Table 1). In the early AR group (age at AR ≤ 5.1 years) BMI at menarche was similar in the PCOS and control groups (Table 2). However, women with PCOS with early timing of AR had higher BMI and higher WC at ages of 31 and 46 compared with controls with early AR (Table 2, Fig. 4)

**Table 2:** Characteristic in different AR age groups

	Early AR (≤5.1 y)			Normal/late AR (>5.1 y)			
	Control women (*n* = 252)^a^	PCOS (*n* = 85)^a^	*p* ^b^	Control women (*n* = 758)^a^	PCOS (*n* = 104)^a^	*p* ^b^	*p* ^c^
Birth weight	3500 [3200; 3845]	3440 [3150; 3635]	0.105	3460 [3150; 3792]	3250 [2942; 3600]	<0.001	0.028
Age at AP (months)	9.0 [9.0; 9.6]	9.0 [9.0; 9.15]	0.301	9.0 [9.0; 9.0]	9.0 [9.0; 9.0]	0.677	0.798
BMI at AP (kg/m^2^)	17.82 [17.35; 18.30]	17.81 [17.16; 18.31]	0.656	17.73 [17.26; 18.25]	17.55 [17.17; 17.99]	0.083	0.178
Age at AR (years)	4.6 [4.2; 4.6]	4.5 [4.0; 4.8]	0.392	5.9 [5.6; 6.3]	5.9 [5.5; 6.3]	0.053	<0.001
BMI at AR (kg/m^2^)	16.11 [15.52; 16.76]	15.98 [15.27; 17.07]	0.621	15.00 [14.46; 15.54]	14.96 [14.40; 15.51]	0.876	<0.001
Age at menarche (years)	12.0 [11.0; 13.0]	12.0 [11.0; 13.0]	0.715	13.0 [12.0; 14.0]	13.0 [12.0; 14.0]	0.416	<0.001
BMI at menarche (kg/m^2^)	20.42 [19.34; 22.42]	20.52 [19.30; 23.00]	0.551	17.30 [16.37; 18.36]	17.79 [16.68; 18.55]	0.008	<0.001
BMI 31 y (kg/m^2^)	25.40 [23.08; 28.71]	27.10 [23.97; 32.16]	0.009	21.88 [20.28; 23.96]	23.11 [20.82; 24.77]	0.005	<0.001
BMI 46 y (kg/m^2^)	28.80 [24.88; 33.42]	30.19 [26.76; 35.89]	0.019	24.60 [22.15; 27.71]	25.65 [23.09; 29.08]	0.02	<0.001
WC 31 y (cm)	83.00 [76.00; 92.88]	87.00 [79.00; 99.00]	0.016	74.50 [69.25; 81.00]	78.00 [71.00; 87.50]	0.028	<0.001
WC 46 y (cm)	90.75 [82.53; 102.88]	98.00 [87.50; 109.00]	0.014	83.00 [75.50; 92.00]	85.00 [79.00; 94.00]	0.001	<0.001
Testosterone 31 y (nmol/l)	1.0 [0.8; 1.3]	1.4 [0.9; 1.6]	<0.001	1.0 [0.8; 1.3]	1.3 [1.0; 1.8]	<0.001	0.879
Testosterone 46 y (nmol/l)	0.8 [0.6; 1.1]	0.9 [0.6; 1.0]	0.337	0.8 [0.6; 1.1]	0.9 [0.7; 1.1]	0.100	0.371
SHBG 31 y (nmol/l)	51.5 [39.5; 74.4]	40.9 [23.6; 64.4]	0.001	63.0 [46.6; 83.9]	53.6 [40.8; 76.0]	0.035	0.002
SHBG 46 y (nmol/l)	48.2 [36.4; 68.3]	45.3 [29.7; 61.2]	0.090	55.2 [38.9; 77.2]	51.2 [37.4; 72.8]	0.264	0.074
FAI 31 y	1.84 [1.32; 2.62]	3.16 [1.85; 5.16]	<0.001	1.57 [1.12; 2.13]	2.21 [1.29; 3.50]	<0.001	0.012
FAI 46 y	0.02 [0.01; 0.02]	0.02 [0.01; 0.03]	0.020	0.02 [0.01; 0.02]	0.02 [0.01; 0.03]	0.007	0.334
cFT 46 y (nmol/l)	0.011 [0.008; 0.014]	0.012 [0.010; 0.015]	0.029	0.010 [0.008; 0.014]	0.012 [0.008; 0.015]	0.005	0.659

Adiposity rebound is considered here early when occurring at age 5.1 or younger.

Reported as mean ± SD or median with (25%; 75% quartiles) and as percentiles (%). Significance tests for continuous variables were performed using independent samples *t*-test or Mann–Whitney *U* test as appropriate

*AP* adiposity peak, *AR* adiposity rebound, *BMI* body mass index, *WC* waist circumference, *SHBG* sex hormone-binding globulin, *FAI* free androgen index, *cFT* calculated free testosterone, *PCOS* polycystic ovary syndrome

^a^The numbers may vary in different analysis due to some missing data.

^b^Compared with controls in the same AR group

^c^Women with PCOS with early AR compared with women with PCOS in normal/late AR group

### Early AR and androgen levels later in life

Regardless of timing of AR, women with PCOS had higher levels of T, lower levels of SHBG, and higher FAI/cFT, and no significant correlation between AR and T was found at age 31 (*r* = −0.07, *p* = 0.401) or 46 (*r* = 0.06, *p* = 0.504) (Table 1, Supplementary Fig. 2). Moreover, T at age 31 or 46 was not an independent risk factor for timing of AR.

## Discussion

In this prospective population-based cohort study we investigated for the first time the associations between birth weight, early growth, and AR timing vs. PCOS diagnosis and BMI by age 46. Here we report lower birth weight and a differential growth pattern during childhood in women with PCOS. BMI growth trajectories in the PCOS group started to deviate from those in control women early on. In women with PCOS AR occurred earlier, and mean BMI remained higher thereafter until the age of 46. In our study population, an AR drop of 1 year was associated with a 1.62-fold greater OR for PCOS diagnosis by age 46 and the risk was independent of maternal pre-pregnancy BMI, maternal smoking, gestational age, BMI, WC, and serum testosterone levels. Furthermore, women with PCOS and early AR presented with higher BMI and WC at age 31 and/or 46 years compared with controls with early AR. Interestingly, serum testosterone levels at age 31 or 46 were not associated with timing of AR, even though hyperandrogenism has been associated with a more severe BMI outcomes in women with PCOS.

Previous studies have shown that low birth weight in children associates with adverse metabolic outcomes and increased cardiovascular disease-related mortality later in life [3, 4]. In the present study, women with PCOS had a lower birth weight compared with controls, and the results of several [15, 19] but not all [20, 21] studies are in line with our findings. However, the association between birth weight and PCOS diagnosis by age 46 was relatively weak, most likely reflecting the fact that several factors affect birth weight in general. In addition we did not observed difference between group for clinically defined low birth weight or SGA. Moreover, even though some investigators have reported a higher rate of prematurity in the offspring of women with PCOS [22], in the present data there was no difference in gestational ages. Unfortunately, the data on possible PCOS status of the mothers were not available. Given that birth weight is sensitive to several pregnancy-related genetic and environmental factors, recent study have also suggested that it may not share the same programming with postnatal growth, especially the growth in childhood [23].

Early timing of AR has been shown to be associated with adverse metabolic outcomes, such as obesity, higher triglyceride and low-density lipoprotein-cholesterol levels, insulin resistance, and metabolic syndrome in adulthood but also in children shown as early as at 7 years of age [5, 6, 24, 25]. Similarly, PCOS has been associated with many of these metabolic abnormalities, with some studies showing weight gain from adolescence to adulthood being a risk factor for PCOS [9]. However, it has been suggested that pre-pubertal time is critical for the development of PCOS, with some reports showing that early life obesity with insulin resistance may be attributed to later development of PCOS [11, 26]. Indeed, in the present data set, childhood BMI growth in cases of PCOS appeared to deviate from that in the controls early on. The AR occurred earlier (5.19 vs. 5.60 years), and the BMI at AR was higher in women with PCOS. From AR onwards women with PCOS had higher BMIs at menarche and at ages 31 and 46 similarly to what has been found in previously reported studies in non-PCOS populations [5]. Indeed, early AR, occurring before the age of 5.1 years in cases of PCOS appeared to be associated with higher BMI and WC at ages 31 and 46 in comparison with the situation in control women experiencing early AR, suggesting a more severe metabolic phenotype in these women. Interestingly, the women with OA + H presented with even earlier AR timing compared to less metabolically compromised women with srPCOS. Importantly, given that there were also non-PCOS women with early AR and adverse BMI outcomes later in life, early timing of AR was associated with PCOS diagnoses also after adjusting for BMI. These findings further strengthen the hypothesis that early childhood is a sensitive period for not only early metabolic risks but also for PCOS [11, 12, 25, 26]. It is tempting to speculate that early timing of AR is the first sign of a developmental process towards PCOS possibly reflecting the early predisposition to this condition triggered by the environmental factors.

Given that hyperandrogenic women with PCOS commonly present with the highest BMI [27], we assessed the association between serum testosterone concentrations at ages 31 and 46, and AR timing. In general, there were no correlations between serum testosterone or FAI and timing of AR, and women with PCOS were hyperandrogenic compared with controls regardless of the timing of AR. Despite this finding, androgen levels during childhood have been shown to be associated with the timing of AR, since children with premature adrenarche or 21-hydroxylase deficiency do have early AR [28, 29]. Regarding 21-hydroxylase deficiency, one study hypothesized that low birth weight with concomitant excess androgen exposure during the fetal and perinatal periods may contribute to timing of AR, whereas in cases of premature adrenarche it is thought that early growth acceleration leads to obesity that may results into premature adrenarche [28, 29]. To support this, previous studies have revealed high testosterone levels in obese adolescent girls [27], but unfortunately, data on serum testosterone levels in adolescence were not available in our study cohort. Thus, the role of childhood androgen levels in connection with AR timing in cases of PCOS still remains unsolved.

This study has several strengths but also some limitations. The NFBC provides unique longitudinal data for studying early BMI trajectories and hormonal and metabolic outcomes later in life in women with PCOS. The participation rate has been high throughout all data collection points and the early BMI growth data were obtained from the health register of a child health program that is available and free for all families. To our knowledge, this is the first study carried out to investigate BMI trajectories in women with PCOS from early infancy to premenopausal age in a population-based setting, with the additional benefit of having the possibility to adjust the data for several confounding factors. The fact that gestational ages were not determined by means of ultrasonography may have had some effect on the detection of subtle differences between the study groups. Furthermore, despite the high genetic predisposition related to PCOS, the mothers in the present data set were not screened for PCOS. Thus, the adverse pregnancy outcomes reported earlier in cases of PCOS, including low birth weight and the risk of an LGA or SGA infant, may not apply here [22]. The limitations also include self-reported PCOS symptoms and diagnoses. However, we have previously shown the validity of self-reported symptoms as reliable indications of PCOS [9, 10, 16]. The study included only Caucasians; thus, the results may not apply to different ethnicities or populations with higher childhood obesity rates.

## Conclusions

The results of the present study indicate that early AR is one of the risk factors of PCOS, independent of BMI, and it appears to result in more adverse BMI outcomes in adulthood in cases of PCOS than in controls with early AR. As the BMI trajectories appear to deviate early on from the time of AR onwards, early AR may be the first sign of a developmental process toward a PCOS phenotype. More studies are needed to shed light on the prenatal triggering mechanisms behind PCOS in humans.

## Supplementary information

Supplementary Figure 1

Supplementary Figure 2

Supplementary Figure 3

Supplementary legends

APC form
