# Supplementary figures and images for: Age at adiposity rebound in childhood is associated with PCOS diagnosis and obesity in adulthood—longitudinal analysis of BMI data from birth to age 46 in cases of PCOS
Source: Int J Obes (Lond). 2019 Feb 4;43(7):1370–9. doi: 10.1038/s41366-019-0318-z (PMC6760596; doi:10.1038/s41366-019-0318-z)

# Northern Finland Birth Cohort 1966

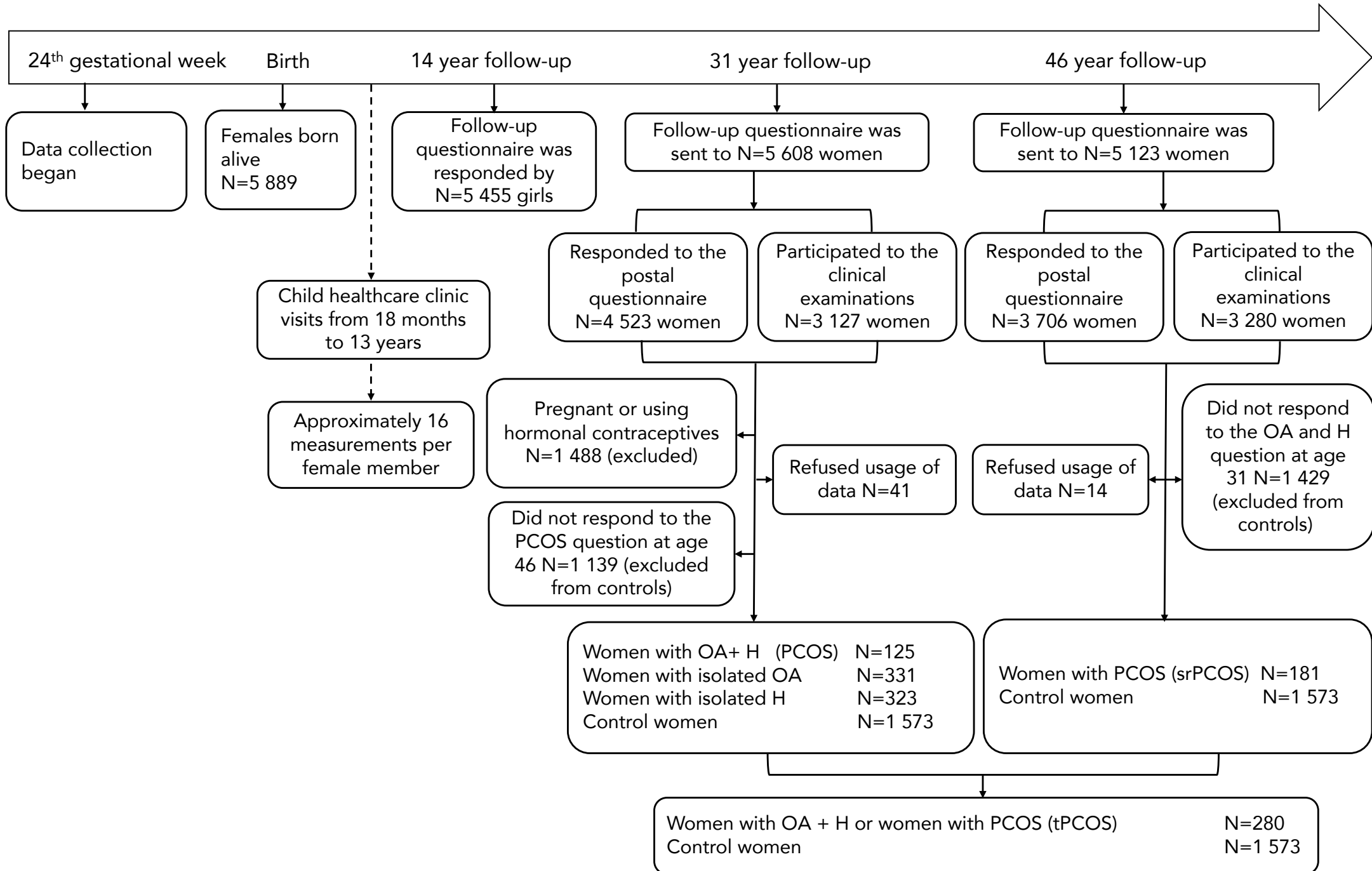

Supplement: Supplementary file 1 — Supplementary Figure 1 [file 41366_2019_318_MOESM1_ESM.pdf]

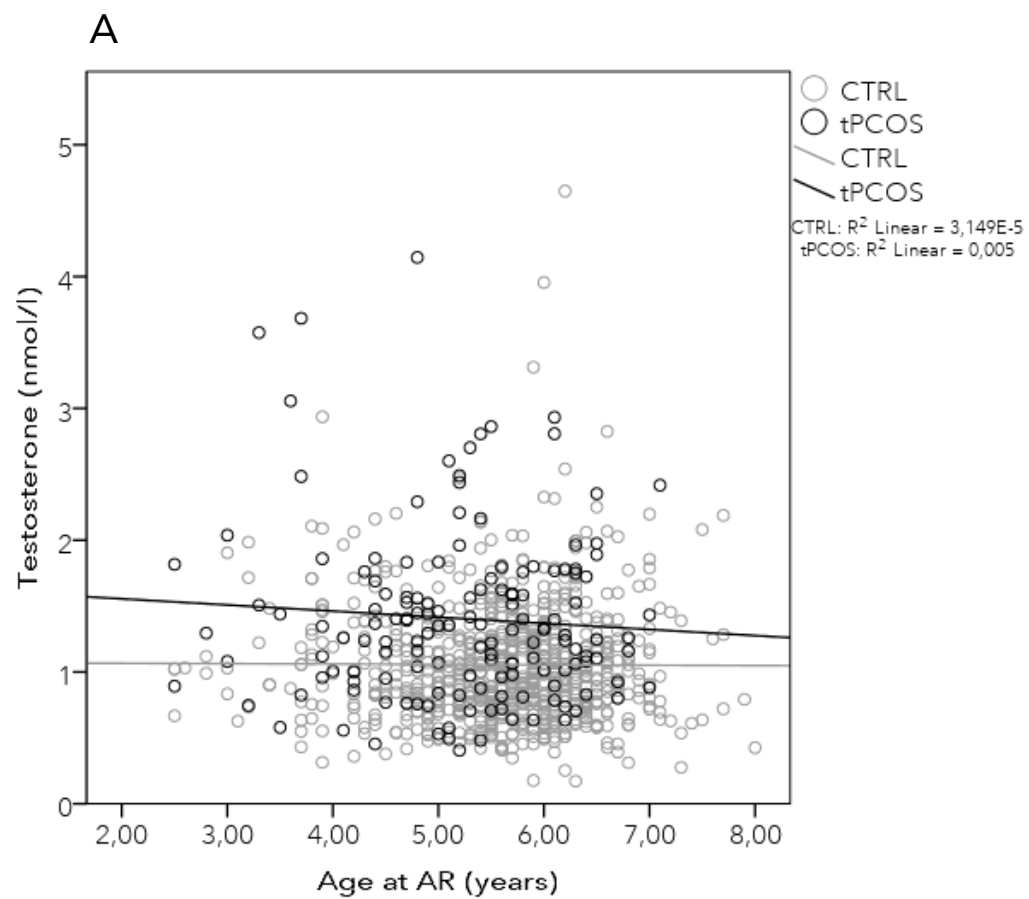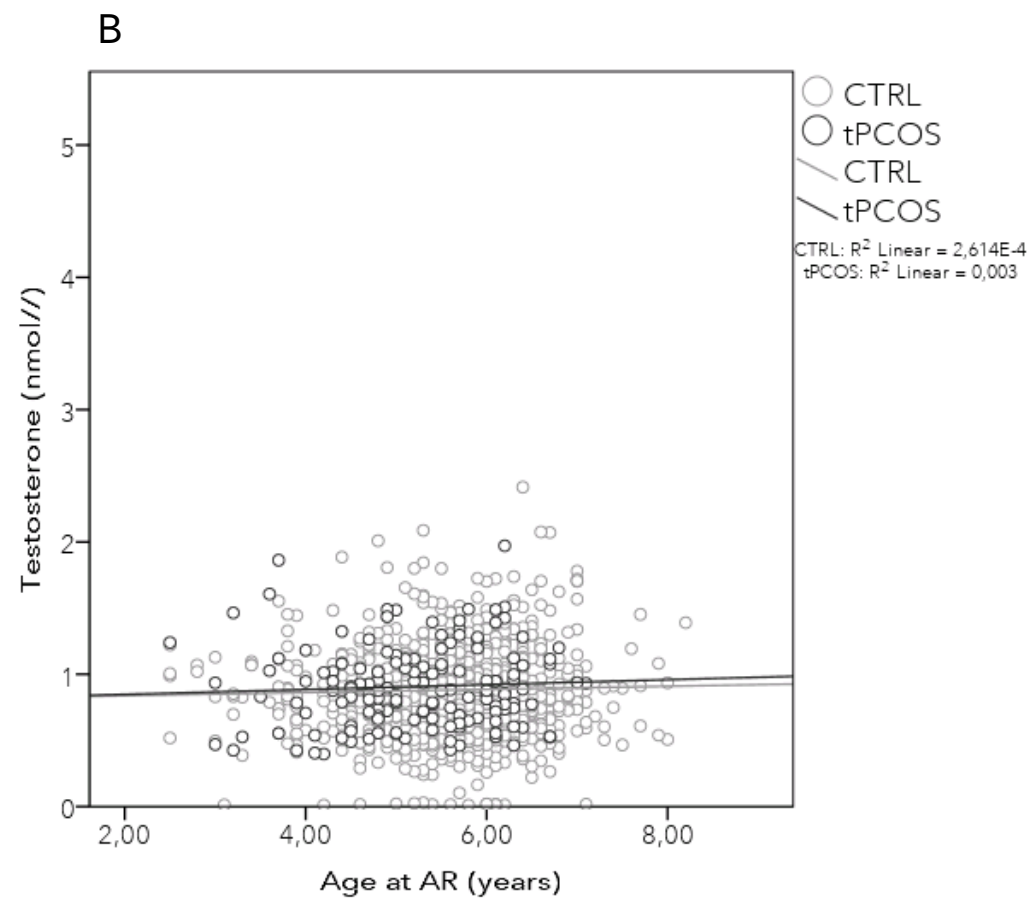

Supplement: Supplementary file 2 — Supplementary Figure 2 [file 41366_2019_318_MOESM2_ESM.pdf]

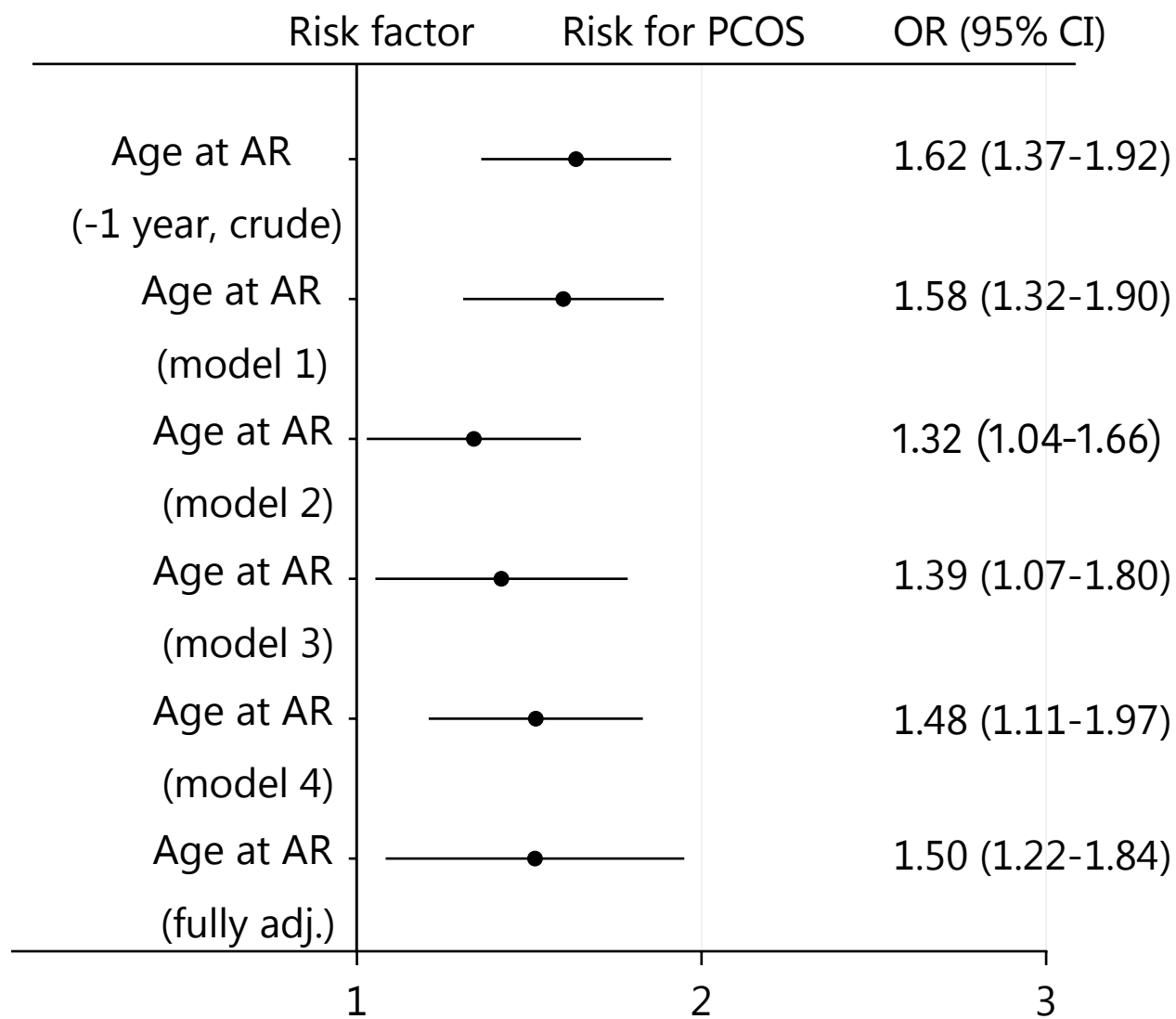

Supplement: Supplementary file 3 — Supplementary Figure 3 [file 41366_2019_318_MOESM3_ESM.pdf]

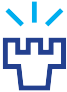

UNIVERSITY OF OULU

Supplement: Supplementary file 5 — APC form [file 41366_2019_318_MOESM5_ESM.pdf]
