# Supplementary material for: Age at adiposity rebound in childhood is associated with PCOS diagnosis and obesity in adulthood—longitudinal analysis of BMI data from birth to age 46 in cases of PCOS
Source: Int J Obes (Lond). 2019 Feb 4;43(7):1370–9. doi: 10.1038/s41366-019-0318-z (PMC6760596; doi:10.1038/s41366-019-0318-z)
Supplement: Supplementary file 4 — Supplementary legends [file 41366_2019_318_MOESM4_ESM.docx]

**Supplementary Figure 1.** Flowchart of the study population.

**Supplementary Figure 2**. Correlation between adiposity rebound (AR) timing and testosterone levels at age 31 (A) and at age 46 (B) in women with PCOS (tPCOS) and in control women (CTRL). No correlation was observed between the timing of AR and serum testosterone levels at age 31 or 46.

**Supplementary Figure 3.** Risk factors associated with PCOS diagnosis by age 46. Different adjustment models described below. The result are expressed, for birth weight: by 100g decrease in birth weight and for AR per 1 year decrease in the age at AR. Early timing of AR remained a significant risk factor of PCOS diagnosis even after adjusting for confounding factors. We used logistic regression analysis and the results are reported as odds ratios (ORs) with 95% confidence intervals (95% CIs).

Birth weight adjusted for maternal pre-pregnancy BMI, maternal smoking and gestational age.

Model 1: Age at AR + maternal pre-pregnancy BMI, maternal smoking, gestational age

Model 2: Model 1 + BMI

Model 3: Model 1 + WC

Model 4: Model 1 + T at 31y

Model 5: Model 1 + BMI + WC+ T at 31y
